# Chemical Profiling of Volatile Organic Compounds in the Headspace of Algal Cultures as Early Biomarkers of Algal Pond Crashes

**DOI:** 10.1038/s41598-019-50125-z

**Published:** 2019-09-25

**Authors:** Kristen L. Reese, Carolyn L. Fisher, Pamela D. Lane, James D. Jaryenneh, Matthew W. Moorman, A. Daniel Jones, Matthias Frank, Todd W. Lane

**Affiliations:** 1 Biosciences and Biotechnology Division, Physical & Life Sciences Directorate, Lawrence Livermore National Laboratory, Livermore, CA 94550 USA; 2 Department of Chemistry, Michigan State University, East Lansing, MI 48823 USA; 3 Systems Biology Department, Sandia National Laboratories, PO Box 969, Livermore, CA 94551 USA; 4 Nano and Micro Sensors Department, Sandia National Laboratories, PO Box 5800, MS 0892, Albuquerque, NM 87185 USA; 5 Department of Biochemistry and Molecular Biology, Michigan State University, East Lansing, MI 48823 USA

**Keywords:** Small molecules, Applied microbiology, Biodiesel

## Abstract

Algae ponds used in industrial biomass production are susceptible to pathogen or grazer infestation, resulting in pond crashes with high economic costs. Current methods to monitor and mitigate unhealthy ponds are hindered by a lack of early indicators that precede culture crash. We used solid-phase microextraction (SPME) coupled with gas chromatography-mass spectrometry (GC-MS) to identify volatiles emitted from healthy and rotifer infested cultures of *Microchloropsis salina*. After 48 hours of algal growth, marine rotifers, *Brachionus plicatilis*, were added to the algae cultures and volatile organic compounds (VOC) were sampled from the headspace using SPME fibers. A GC-MS approach was used in an untargeted analysis of VOCs, followed by preliminary identification. The addition of *B*. *plicatilis* to healthy cultures of *M*. *salina* resulted in decreased algal cell numbers, relative to uninfected controls, and generated *trans*-β-ionone and β-cyclocitral, which were attributed to carotenoid degradation. The abundances of the carotenoid-derived VOCs increased with rotifer consumption of algae. Our results indicate that specific VOCs released by infected algae cultures may be early indicators for impending pond crashes, providing a useful tool to monitor algal biomass production and pond crash prevention.

## Introduction

As the energy needs of the world increase, dependence on non-renewable sources of energy remains a concern. Increased production of corn starch or sugarcane-based ethanol has resulted in increased atmospheric carbon dioxide levels, diversion of arable land from food production, and increased consumer cost for sugar and corn^1^. For these reasons, microalgae production systems are considered a promising avenue for biofuel production. Microalgal strains are capable of growth in a range of environments (e.g. freshwater, marine, hypersaline, highly acidic) including high-nutrient municipal wastewater systems^2^, allowing for simultaneous bioremediation and biofuel production. Microalgae’s ability for rapid growth in non-potable (brackish or marine) water sources using non-arable land, combined with their high capacity for fixation of atmospheric carbon dioxide, and high lipid-to-biomass ratios are significant advantages toward its use as a biofuel feedstock. The development of optimized systems for sustainable and dependable biofuel production through algal pond systems are necessary as the global energy strategies continue to evolve^3^ (for review, see Kayitar 2017).

**Figure 1:**
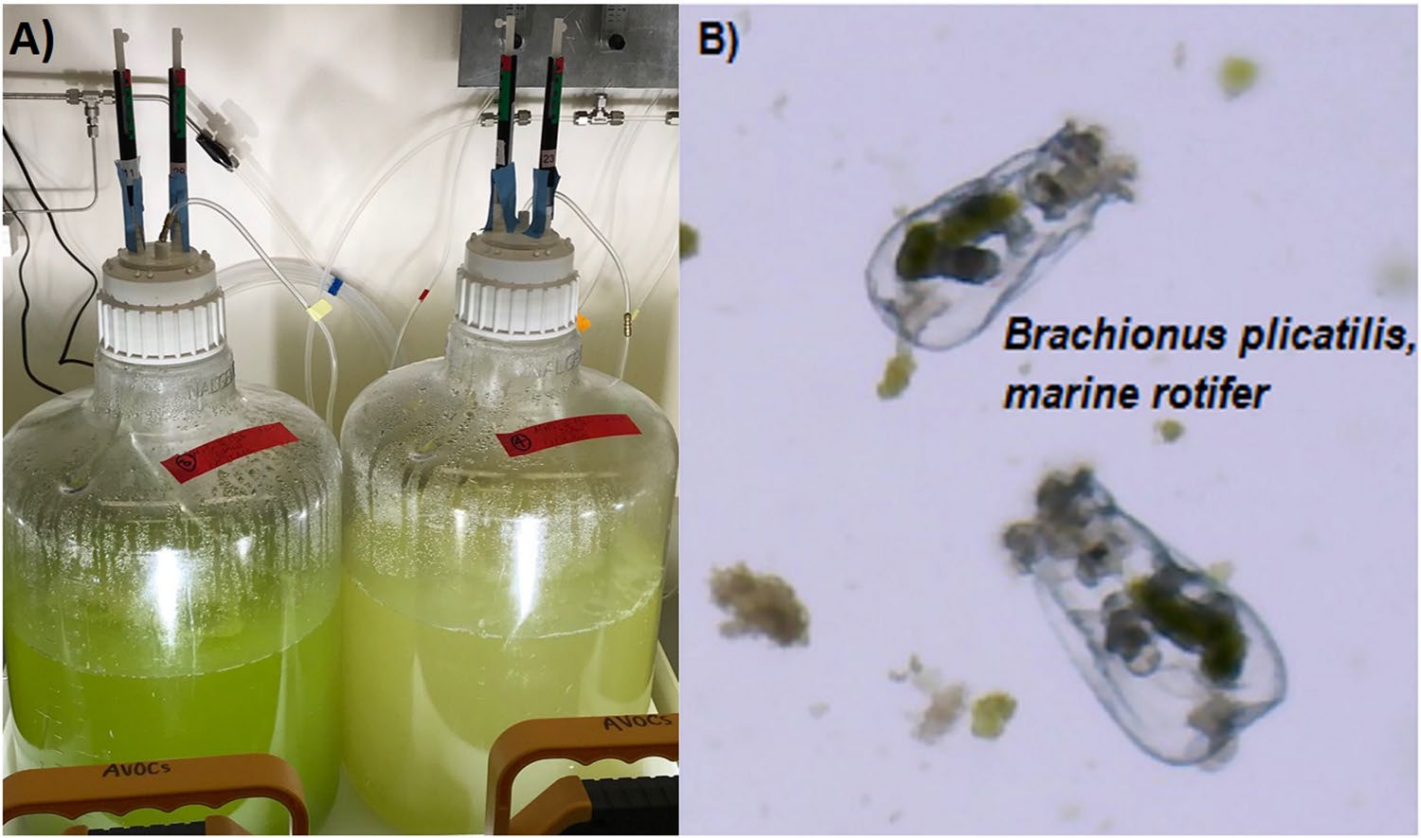
(**A**) 15 L algal mass cultures (analgous to production ponds) with a healthy algal culture on the left compared with a crashed algal culture on the right. (**B**) *Brachionus plicatilis* (average length 160 µm), marine rotifer, in a field of microalgae, *Microchloropsis salina*.

A major challenge faced in algal production are ‘pond crashes’, which are defined as devastating, often unpredictable losses of entire crops due to parasitism, grazing, weather, or many other factors. Closed photobioreactors are less likely to be susceptible to contamination with deleterious species (e.g. viruses, fungi, protozoans, detrimental microbes), but involve higher capital costs^4^ and, once contaminated, can be difficult to disinfect. Open algal ponds are less expensive to set up but are more likely to succumb to crashes (Fig. 1A) caused by grazing or parasitism^5,6^. Notably, a single adult marine rotifer, *Brachionus plicatilis* (Fig. 1B), can consume 200 microalgal cells per minute and double in population within 1–2 days^7^. It is estimated that pond crashes account for 30% loss of annualized algal production^4^ and represent a significant economic barrier to biofuel production^8^.

Current pond crash mitigation strategies, both prophylactic and interdictive, are largely focused on chemical treatments, such as hypochlorite^9^, copper^10,11^, quinine sulfate^12^, rotenone^13^, additives that lower pH (to less than 3.0)^14^, and biocides, such as tossendanin^15^. The use of chemical countermeasures as a prophylactic strategy to prevent pond crashes is prohibitively expensive for most algal industry business models. Chemical additives can degrade from sun exposure and may need to be repeatedly added to cultures to maintain protection. In addition, frequent and repeated chemical application can be environmentally detrimental through the development of resistant pest species or through unacceptable off-target effects (e.g., pisicidal effects of rotenone). However, when applied early and in a targeted fashion after the detection of a deleterious species^16^, chemical additives can be highly effective at saving algal cultures [for review of crop protection strategies, see Fisher & Lane 2019].

In order to attain the production levels of 25 g m^−2^ d^−1^ ash free dry weight (or 2,500 gallons of biofuel per acre per year)^17^, deemed necessary for economic algal biofuel production, cost-effective pond monitoring strategies are necessary to reduce culture loss and increase annualized production. Currently, algal production facilities utilize light microscopy to identify contaminants, pathogens, and competing algal strains that could lead to the demise of the desired algal strain^18^. However, microscopy is slow, labor-intensive, and requires advanced operator training for differentiating various microbiota. Alternative methods involving automated and semi-automated technologies, such as FlowCAM imaging flow-cytometry^19,20^, polymerase chain reaction, and hybridization-based assays^21,22^ are under development to increase sensitivity and expedite analysis for daily algal culture monitoring.

Volatile organic compounds (VOCs) are carbon-containing molecules with high vapor pressures at ambient temperatures^23^. Within the field of chemical ecology, VOCs have been identified as secondary metabolites and include, but are not limited to, pheromones, semiochemicals, odorants, and phytohormones^24,25^. Algal VOC production has been associated with intra- and inter-species communication, allelopathy, semiochemical production, and predator deterrence^26^ [for review, see Zuo 2019]. A well characterized example of an algal volatile involves conversion of nonvolatile dimethylsulfoniopropionate (DMSP) to volatile dimethyl sulfide (DMS). In intact *Emiliania huxleyi* cells, conversion of DMSP to DMS by the enzyme DMSP lyase is minimal. However, during algal grazing, such as by the dinoflagellate *Oxyrrhis marina*, *E*. *huxleyi* is disrupted, releasing DMSP. Once in solution, DMSP lyases, including those from bacteria, catalyze conversion of DMSP to DMS^27,28^. DMS then acts not only as a deterrent against herbivory by *Oxyrrhis marina*^29^, but additionally as an attractant for other species such as birds and fish^30^.

The aims of this study were to 1) develop a methodology to detect VOCs from healthy algal cultures (*Microchloropsis salina*) as well as algal cultures in the presence of a grazer (*M*. *salina* cultures with marine rotifer *Brachionus plicatilis*) and 2) evaluate whether specific VOCs could serve as early indicators of an imminent culture crash. A setup based upon solid-phase microextraction (SPME) fibers coupled with gas chromatography-mass spectrometry (GC-MS) allowed for non-invasive monitoring of volatile emissions. Compounds present during the active grazing period of rotifers on algal cultures, but not produced in healthy controls, were deemed potential biomarkers of high stress conditions. We propose that these biomarker compounds are potential diagnostic tools for chemical monitoring systems in microalgal cultivation systems to enabling the early detection of culture stress for improved algal crop production.

## Results

### Cell counts of infected and control cultures

**Figure 2:**
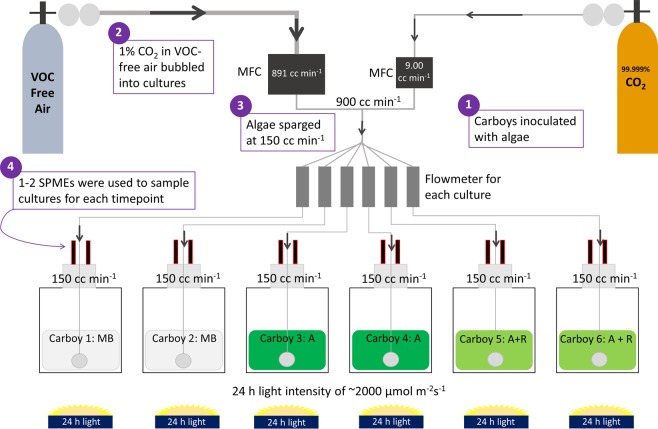
Schematic of experimental setup for growth *M. salina* (Algae, A) in the presence of *B. plicatilis* (Rotifer, R) for 5 d. Mass flow controllers (MFCs) mixed 1% CO_2_ with VOC-free air to sparge 15 L cultures at 150 cc min^−1^. One to two SPME fibers were used to sample the headspace of media blank (MB), Algae only (A), and Algae + Rotifer (A + R) carboys for 30–60 min each at various timepoints over 2–4 days.

**Figure 3:**
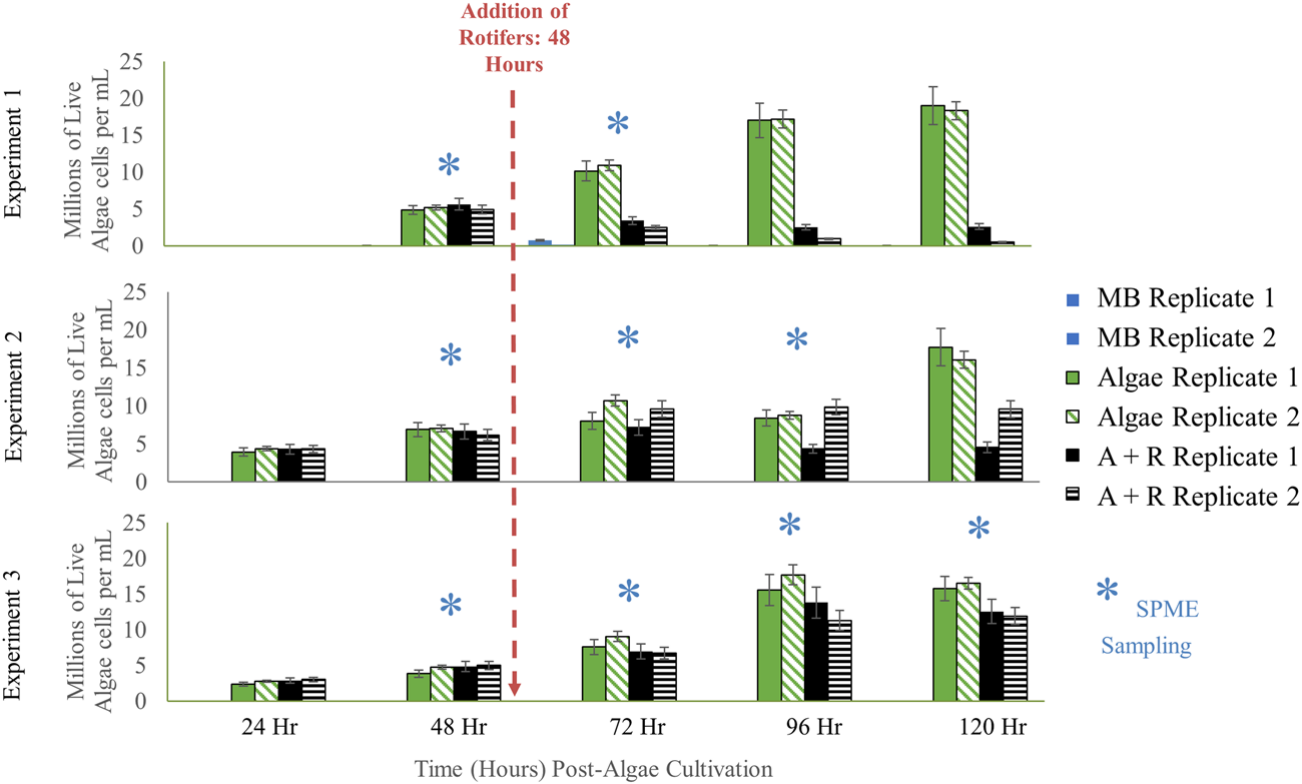
Algae concentration as determined by fluorescence measurements collected for three experiments. Similar coloring and patterns represent biological replicates of each condition: media blanks (MB), Algae (A), and Algae + Rotifer (A + R) cultures. Error bars represent standard deviation derived from duplicate measurements for each sample. Significance levels for conditions that exhibited p < 0.05 are in Supplemental Table 1. Blue asterisks (*) indicate the time points for headspace VOC sampling by SPME fibers.

Our experimental setup (summarized in Fig. 2) facilitated headspace volatile monitoring of *M*. *salina* with and without the microalgal grazer, *B*. *plicatilis*. In each experiment, algal cell concentrations and VOC headspace samples were collected at various time points for *M*. *salina* alone (abbreviated Algae or A), *M*. *salina* and *B*. *plicatilis* (abbreviated Algae + Rotifer or A + R), and ESAW media blanks (abbreviated Media Blank or MB) (Fig. 3, Supplemental Fig. 1). At 48 hours after inoculation, algal concentrations across all cultures were similar, approaching the mid- to late- stages of logarithmic growth. At this time, *B*. *plicatilis* were added to two *M*. *salina* cultures, resulting in time-dependent decreases in algal density relative to controls (Fig. 3). Despite consistent growth conditions, 96 hours after the initial cultures were started and 48 hours after rotifers were added, the Algae + Rotifer cultures displayed different extents of algal biomass loss attributed to rotifer grazing (see Fig. 3). This variation in rates of biomass loss may arise from differences in rotifer lots.

### Headspace VOC results

**Figure 4:**
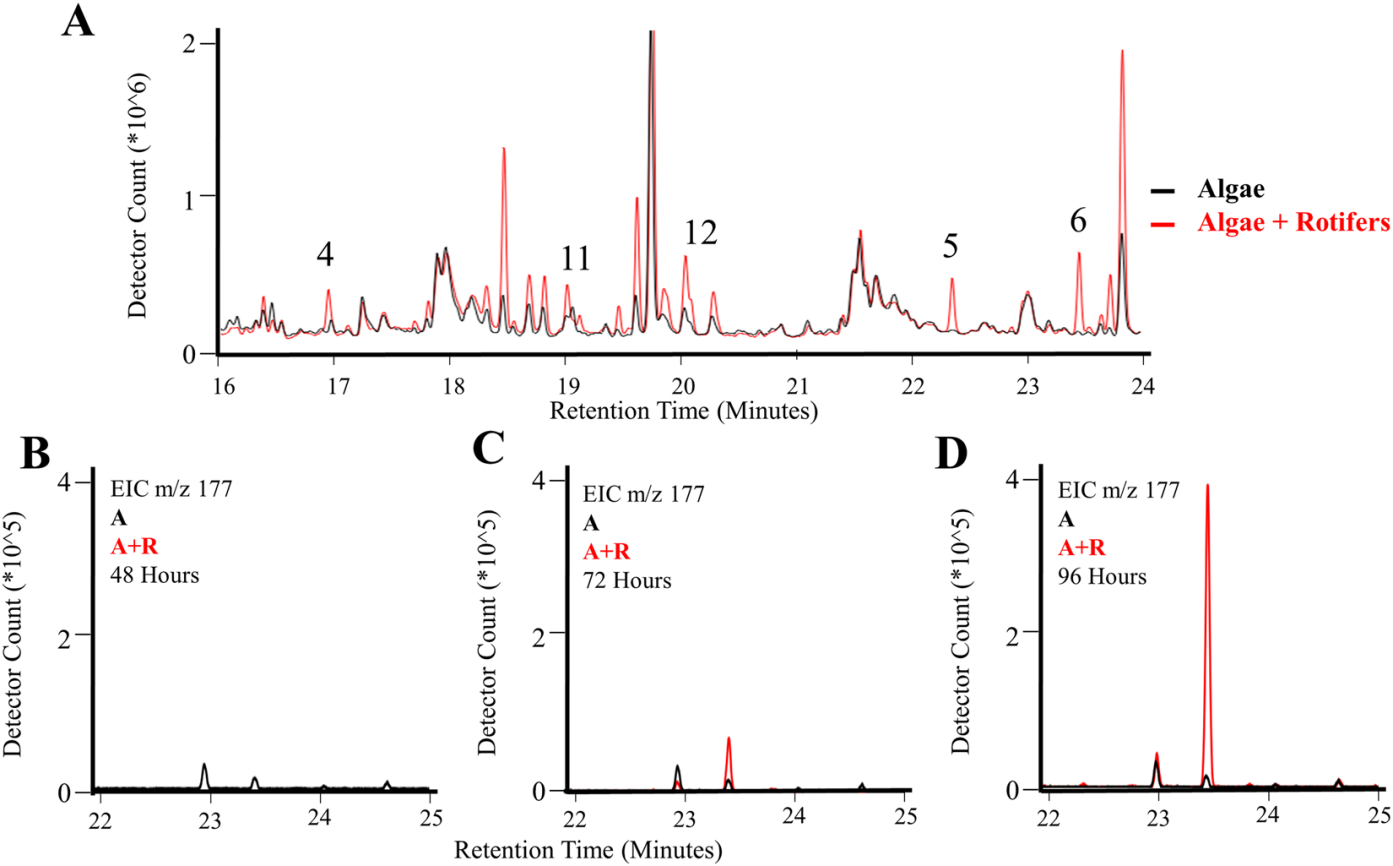
Example chromatograms for observed VOCs sampled from Algae (A) and Algae + Rotifer (A + R) cultures between 16 and 24 min, (**A**) Total ion chromatogram with indicated VOCs (Annotations See Table 1), (**B**–**D**) extracted ion chromatograms monitoring increase in compound 6 over time (*m/z* 177, RI 1495).

Headspace VOCs were sampled with SPME fibers for 30–60 min each at various time points (as indicated in Fig. 3) and analyzed by GC-MS. Qualitative and quantitative differences were observed in the VOC profiles of Algae + Rotifer cultures compared to the Algae cultures. Example total ion chromatograms for Algae and Algae + Rotifer cultures taken approximately 24 hours after addition of rotifers (Experiment #3) are shown in Fig. 4A. Several VOCs that differentiate the two culture conditions are enumeratedi (Fig. 4A, annotations in Table 1) and are potential early indicators of algal grazing or death. Extracted ion chromatograms were utilized to improve visualization of individual VOCs. Figure 4B–D demonstrate the increase in an VOC displaying a base peak *m/z* 177 and retention index 1495, observed over the time course of the experiment in Algae + Rotifer cultures. Although the Algae chromatogram for *m/z* 177 also displays a small peak at the same retention time, this VOC was not detected using the given experimental criteria for data processing.

**Table 1:** VOCs robustly and repeatedly detected from Algae and Algae + Rotifer experiments.

	Compound #	Mass	Tentative Compound Class*	NIST14 ID	NIST % Match	Experimental Retention Index	Theoretical Retention Index	Experiment #		
								1	2	3
VOCs detected in A+R cultures	1	82	Carotenoid	2,2,6-trimethylcyclohexanone	79	1021	1036		X	X
	2	107				1181		X	X	X
	3	121	Phenol			1191			X	X
	4	137	Carotenoid	2,6,6-trimethyl-1-cyclohexene-1-carboxaldehyde	81	1209	1220	X	X	X
	5	121	Carotenoid	4-(2,6,6-trimethyl-1-cyclohexen-1-yl)-2-butanone	76	1419	1433	X	X	X
	6	177	Carotenoid	trans-${\rm{beta}}$-ionone	94	1495	1486	X	X	X
	7	57	Alkane			1691		X	X	X
VOCs detected in A+R and A cultures	8	71				1039			X	X
	9	96	Methyl Ester	3-Nonenoic acid, methyl ester	74	1134	1191	X	X	X
	10	341				1139			X	X
	11	71				1293			X	X
	12	138	Terpene/Carotenoid			1338			X	X
	13	73	Fatty acid (Hexadecanoic acid)			1983			X	X
	14	192				2197			X	X

*Tentative compound class for unknown compounds is based on fragmentation in averaged mass spectra determined via chromatographic deconvolution and alignment.

The number of compounds detected from deconvolution of chromatographic peaks varied with each sample. The analysis of a single sample commonly detected 100–200 chemical compounds, many of which were attributed to background (known from control measurements). Application of chromatographic peak alignment across the data from all samples and at every timepoint generated a list of more than 1800 compounds, consisting of both algal VOCs and extraneous signals from the experimental setup. Many compounds were attributed to known background or were not found reproducibly. Application of the filtering criteria based upon algal abundance and detection frequency across experimental replicates identified the most robust compounds as potential VOC biomarkers from either Algae or Algae + Rotifer cultures, removed irreproducible compounds, and narrowed the extensive list to ~50 compounds in any single experiment. Table 1 shows biomarkers that were only observed across multiple experiments. For a detailed list of the volatile biomarkers detected in each experiment, refer to Supplemental Table 1.

Comparison of three replicate experimental infections of both Algae and Algae + Rotifer cultures revealed several VOCs that were reproducibly observed in 1) Algae + Rotifer cultures and 2) both Algae and Algae + Rotifer cultures, represented in Table 1, despite the different rates in algal biomass loss. For example, Compound 6 monitored in Fig. 4B–D was identified with a 94% confidence score as *trans*-β-ionone using the NIST14 library. Confidence in this identification increases when considering the calculated experimental retention index (RI) of 1495 was within 5% of the literature theoretical value (1486, NIST 14 database). Within the Algae + Rotifer cultures, all of the discriminating VOCs were structurally-related ketones or aldehydes: (a) Compound 6: *trans*-β-ionone [IUPAC name: (*E*)-4-(2,6,6-trimethyl-1-cyclohexen-1-yl)-3-buten-2-one], (b) Compound 4: β-cyclocitral [IUPAC name: 2,6,6-trimethyl-1-cyclohexene-1- carboxaldehyde], (c) Compound 1: 2,2,6-trimethyl-cyclohexanone, and (d) Compound 5: 4-(2,6,6-trimethyl-1-cyclohexene-1-yl)-2-butanone. We propose these compounds as potential biomarkers indicative of algal stress or death. The VOCs observed in both Algae and Algae + Rotifer cultures were most likely algae-derived volatiles universally present in both conditions, with 3-nonenoic acid methyl ester having an adequate confidence for identification (74% spectral match, <5% RI deviation from theoretical value).

For those VOCs that could not be identified using the initial conservative thresholds, the observed spectra and experimental retention indices provided suggestions for their identification. The suggested compound classes for unknown compounds are provided in Table 1, and their experimental mass spectra are included in Supplemental Fig. 1. For example, the mass spectrum of Compound 12 has similar features to those of the identified carotenoids, suggesting a terpenoid structure with a molecular weight of 208 Da. The mass spectrum of Compound 13 contains characteristic ions of hexadecenoic acid including *m/z* 43, 60, 73, 129, 213, and 256 (M^+^), and its experimental RI is within 1% of the literature RI of hexadecanoic acid (1968).

### Abundance of VOCs

**Figure 5:**
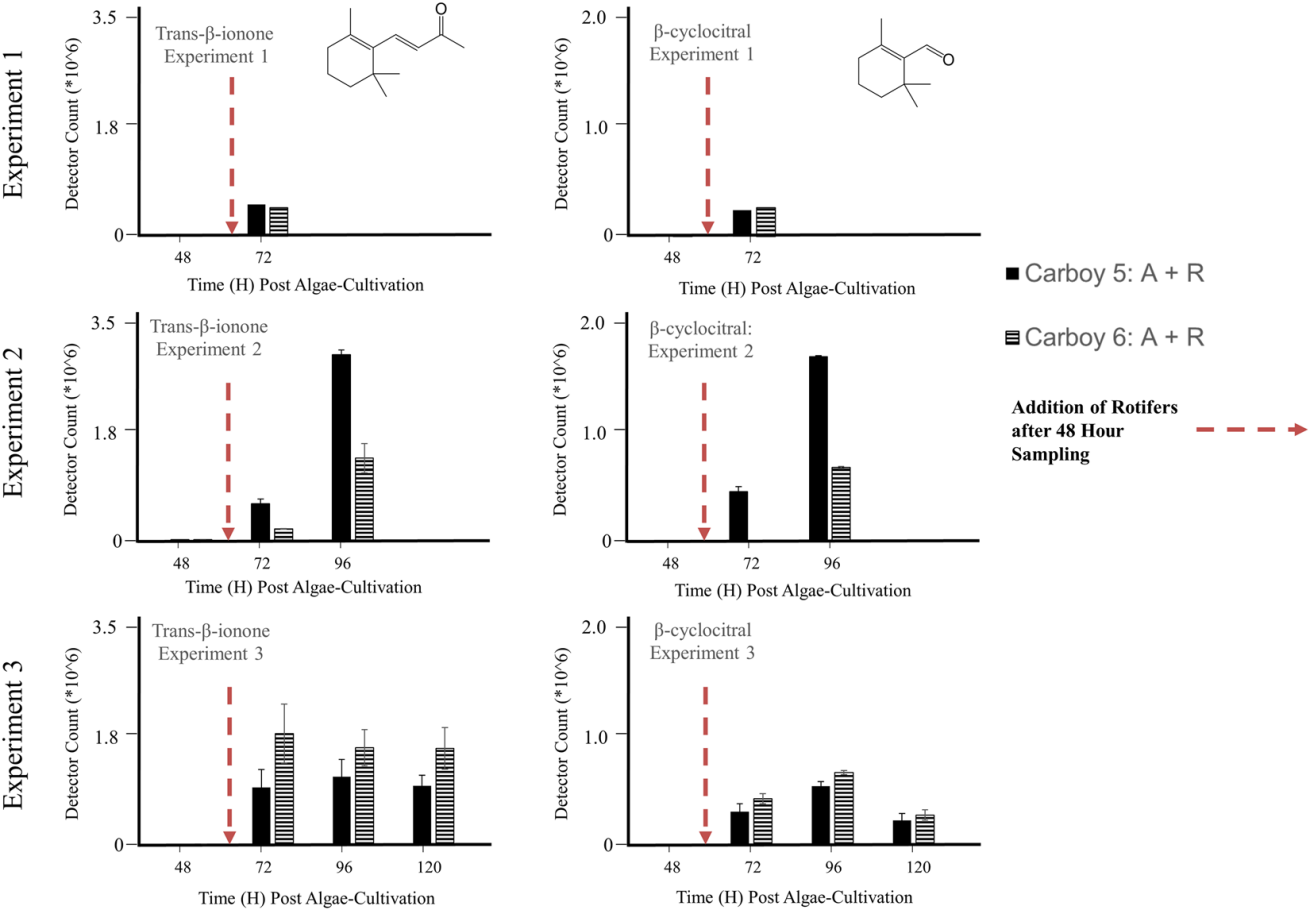
Peak areas of extracted compound chromatograms for trans-β-ionone and β-cyclocitral across Experiments 1, 2, and 3, separated by biological replicates. Error bars represent standard deviation derived from duplicate measurements for each sample. The exposure time for SPME fibers was 30 minutes in Experiment 1 and 60 minutes in Experiments 2 and 3.

In addition to the qualitative analysis, we examined the relationship between rotifer duration of feeding and the abundance of Algae + Rotifer distinguishing VOCs. Levels of Compounds 4 and 6 were compared for individual rotifer cultures (Carboys 5 and 6) across individual experiments (Fig. 5). While there was no detected signal from either compound before the addition of rotifers (48 hours), all detected signals after rotifer addition exceeded 2.0 × 10^5^ counts, 2–3 orders of magnitude above detection threshold. Another carotenoid oxidation product, β-cyclocitral, appeared in Experiment 2 after 24 hours of rotifer feeding, with the signal increasing to more than 6.0 × 10^5^ counts after 48 hours of rotifer feeding. Similar comparisons for Compounds 1–7 are included in Supplemental Fig. 2. Of note, comparison of Fig. 5 to Fig. 3 reveals several instances where these VOCs were detected in Algae + Rotifer cultures before biomass loss was apparent as compared to algae controls. For example, the second biological replicate of Algae + Rotifers in experiment 2 did not differ in algal density from the healthy controls at the 72 h and 96 h timepoints. However, the signals for Compounds 4 and 6 were already large (6.0 × 10^5^ and 1.0 × 10^6^ counts, respectively).

## Discussion

**Figure 6:**
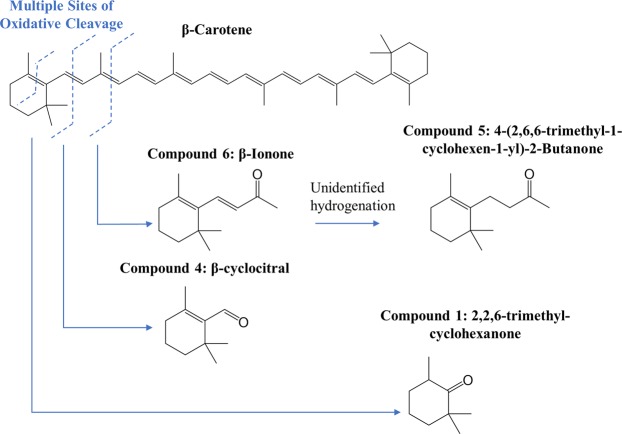
VOCs identified from the headspace of Algae + Rotifer cultures formed from the oxidative cleavage of the carotenoid β-carotene. Only those oxidation cleavages relevant to this study are pictured, but all double bonds across the β-carotene backbone are cleaved.

Our SPME-GC-MS analysis has identified seven discriminating VOCs in *M*. *salina* cultures in the presence of actively-grazing *B*. *plicatilis* (Algae + Rotifer). The absence of these volatiles in the time-matched Algae control cultures (Fig. 3) suggests these chemicals are specific signals of algal grazing or algal death. Many of these chemicals were detected within 24 hours after rotifer addition and before algal cell densities changed substantially (Figs 2, 4). Specifically, Compounds 4, 5, 6, and 7 were identified as early and robust grazing signals observed in *M*. *salina* cultures containing rotifers. Several identified biomarkers – Compounds 1, 4, 5, 6 and 7 – were detected only during rotifer grazing and contained structural similarities, hinting at a shared metabolic pathway. Many of these compounds (Table 1) are known products of carotenoid oxidation^31,32^. Carotenoid-derived substances have been previously observed in microalgal volatile research, largely associated with investigations of flavor or smell components in food production. Carotenoids have important physiological functions as a component of photosystems – the light-harvesting complexes that transfer light energy to chlorophyll^33^. Oxidative cleavage of the carotenoid backbone can occur through enzymatic (carotenoid cleavage dioxygenases) or non-enzymatic (light, oxygen, temperature) mechanisms^33^. Potential pathways for oxidative cleavage of the carotenoid β-carotene are shown in Fig. 6. In our work, the carotenoid-derived VOCs could be generated from the oxidation of β-carotene released upon lysis of *M*. *salina* cells during the digestive process of *B*. *plicatilis*. This would be in agreement with studies of *Arabidopsis* plants exposed to reactive oxygen species resulting in the subsequent release of β-ionone and β-cyclocitral^34^. The results from vascular plants suggest that carotenoid degradation products may be more general indicators of stressed or wounded algae cultures, not solely limited to the interaction of algae with rotifers.

Although there are no reports of such analyses of *M*. *salina*, a small number of studies have examined algae from the genus *Nannochloropsis*, of which *M*. *salina* is a close relative^35^. Van Durme *et al*.^36^ investigated the volatile composition of five microalgae species (*Botryococcus braunii*, *Rhodomonas*, *Tetraselmis* sp., *Nannochloropsis oculata* and *Chlorella vulgaris*) by heating samples (40 °C) to produce volatile signatures under heat stress conditions. Products of carotenoid oxidation, including α- or β- ionone and β-cyclocitral, were identified in all species tested. Interestingly, *N*. *oculata* contained a large abundance of ethanol, 2-hydroxy-2-butanone, and benzaldehyde, while only small amounts of β-cyclocitral and ionones were detected. Hosoglu^37^ likewise characterized the volatilomes of several microalgae species using SPME-GC-MS and GC-olfactometry for both chemical profiles and olfactory properties to benefit incorporation into food products and to minimize unpleasant smells. The species *C*. *vulgaris*, *C*. *protothecoides*, and *T*. *chuii* reportedly contain distinguishing amounts of the carotenoid degradation products, α- and β-ionone and 6-methyl-5-hepten-2-one, while expressing a woody smell.

While VOCs have been observed in analyses of chemical compositions of algae in destructive manners (i.e. heating, sonication, solvent extraction etc.), there are fewer reports of volatiles emitted from live, actively-growing cultures. A variety of live algae-derived volatiles (terpenoids, aldehydes, halogenated compounds, etc.) have been shown to influence the odor quality of water^26^. Zhou *et al*.^38^ investigated changes in the volatilome of intact algae over different growth phases (logarithmic, stationary, and decline phase) for six microalgae (*Thalassiosira weissflogii*, *Nitzschia closterium*, *Chaetoceros calcitrans*, *Platymonas helgolandica*, *Nannochloropsis* spp. (*NMBluh014*-*1*), and *Dicrateria inornate*). The *Nannochloropsis* volatilome was largely dominated by alkanes and alkenes and 8-heptadecene, but no carotenoid by-products were reported. Several functions for actively-released VOCs, including carotenoids, have been postulated, such as tolerance of light and oxidative stressors, signaling the presence of predators^33^, and transfer of information throughout algal colonies^26^. β-cyclocitral has previously been reported as a volatile emitted by the bloom-forming cyanobacterium *Microcystis* as a defense mechanism against grazing by *Daphnia magna*^39^. Similarly, our work focused on the volatiles generated from active grazing of algae, which will generate more rapid algal death compared to natural growth cycles. Here, we confirmed the importance of β-cyclocitral as an indication of algal cell damage due to grazing. Future work will determine if VOCs produced by *M*. *salina* in the presence of *B*. *plicatilis* has a similar role in algal-defense as observed previously.

Using a non-invasive, non-destructive sampling and analysis technique, we have demonstrated that VOCs from the headspace of algae cultures can distinguish between algae cultures with grazing rotifers present and uninfected algal cultures, and may serve as general indicators of algal cell death. In order to discover and validate additional diagnostic markers of grazer infection or other incipient crashes, more extensive study of emitted volatiles from microalgal species is required. For example, low levels of grazer-associated VOCs in healthy algal cultures may result from background rates of algal death. Such background signals are likely modulated by physiological state of the culture (e.g. exponential growth or stationary phase) or by nutrient limation. Thus, an improved understanding of the threshold biomarker concentrations that indicate the need for interdictive treatment is paramount.

It is also worth noting that the list of biomarkers reported in this analysis may be considered conservative owing to the stringency of our filtering criteria. Additional biomarkers may be observed with less stringent parameters for the filtration of detected VOC peaks in data processing. However, the identified carotenoid breakdown products served as indicators for algal crashes across all three experiments. Additionally, our SPME-GC-MS methodology is expected to have broader applications. Complex systems-level dynamics between algae, commensal bacteria, and various grazers will require more sophisticated sampling procedures alongside volatilomics data to include biological data sets, such as transcriptomics, metagenomics, and metabolomics. These systems-level analyses and bioinformatics analysis would be more likely to elucidate biological interactions or implications for the chemicals observed in the volatilome. Non-invasive and non-destructive VOC sampling is an attractive, analytical way to better understand and predict the health of microbial cultures.

## Conclusions

Our work aims to increase the breadth and depth of reported algal and rotifer-specific VOCs, providing a tool to better define the physiological state of microalgal ponds and facilitate greater algal biomass production. A SPME-GC-MS methodology for non-invasive and non-destructive sampling of *M*. *salina* infected by *B*. *plicatilis* aided our discovery of seven putative culture crash biomarkers, including trans-β-ionone and β-cyclocitral, over several timepoints during active crashing of algal ponds. These biomarkers were not detected in cultures displaying natural background levels of cell death, suggesting that these signals are produced by high stress conditions, such as rotifer grazing. Finally, these biomarkers offer potential as diagnostic tools to signal the need for crash mitigation strategies, as several signals were detectable before cell death was evident from changes in cell density. Both VOC baselines and signatures from multiple healthy and infected cultures will be compiled in a data base. Early use of this technique would then include surveilling for the emergence of targeted VOC biomarkers of algal distress or injury above healthy baseline thresholds that are indicative of imminent culture failure.

We envision the use of VOC based monitoring in open ponds and we are in the process of extending this work to such systems. In such environments, one must take into account that the volatile “headspace” of open ponds may be influenced by external sources (e.g. VOCs from the environment, wind effects, particulates, etc.), creating a variable background that would require correction for the levels of biomarker compounds. It is possible to temporarily create closed “headspace” above an area of open pond (perhaps using a large funnel), during sample collection, which would serve to limit outside “noise”.

The SPME fibers used in this experiment are field deployable and can easily be adapted to an algal pond production system. Although SPME-GC-MS has proven powerful for untargeted discovery of algal volatile chemical signatures from healthy or grazed cultures, the cost of state-of-the art laboratory-based GC-MS systems and analyses efforts is prohibitive for using this method for continuous monitoring of industrial scale, open algal ponds. Knowledge gained and biomarkers annotated from our untargeted discovery efforts may guide development of targeted, lower-cost, field-deployable detectors capable of monitoring for changes in diagnostic chemical signatures and detecting volatile signals of infection in real-time to facilitate the timely deployment interdictive strategies to prevent pond crashes. Miniaturized GC-MS systems^40,41^ for field deployable detector systems is one such technology currently under development and optimization^42^ for this type of application.

## Methods

### Axenic microalgae culture

*Microchloropsis salina* (CCMP 1776) was obtained as an axenic stock culture (as determined by the supplier) from the National Center for Marine Algae and Microbiota (NCMA at Bigelow Laboratory, ME, USA). *M*. *salina* cultures were grown as previously described in Fisher *et al*., 2019^43^. For volatilomics experiments, ESAW medium was modified to contain 7.5 mM NaNO_3_, 0.5 mM Na_2_PO_4_. Algal cultures were grown in 15-L of medium in 20-L polycarbonate carboys at room temperature (RT) of ~ 22 °C with 24-h light intensity of ~200 µmol m^−2^s^−1^ for 5 d. Carbon dioxide gas, research purity 99.999% (Matheson Tri-Gas, NJ, USA), and research grade air (70:30 N_2_/O_2_), VOC Free (Matheson Tri-Gas, NJ, USA) were supplied to all samples via two mass flow controllers (one for CO_2_ and one for air). The two mass flow controllers (Alicat, AZ, USA) were set to deliver 1% CO_2_ (9.00 cc min^−1^) and 99% air (891 cc min^−1^), for a total mass flow of 900 cc min^−1^ split equally across six culture vessels (150 cc min^−1^ sparging rate for each sample).

### Xenic marine rotifers

Lots of 10–15 × 10^6^ live, xenic, marine rotifers, *Brachionus plicatilis*, were obtained from Reed Mariculture, CA, USA, 1–2 days before each inoculation and were shipped overnight on ice. Upon arrival, *B*. *plicatilis* were kept at 4 °C until concentrated and inoculated into algal culture for each experiment.

### Preparation of cultures

Experimental cultures were grown in in 20-L polycarbonate carboys (ThermoFisher Scientific, MA USA) containing 15 L nutrient enriched ESAW medium, described above. Cultures were initially inoculated with *M*. *salina* culture to a final concentration of 4–5 × 10^6^ cells mL^−1^ in 15 L. Cultures were continuously sparged at of 150 cc min^−1^ with 1% CO_2_ in air through an air stone bubbler.

After 48 hours of *M*. *salina* growth and acclimation to culturing conditions, 1.32 × 10^6^ live rotifers (final concentration of 88 rotifers mL^−1^) were added to two of the four algal cultures. *Brachionus plicatilis* were allowed to warm for 1–3 h to room temperature (22 °C), were gently concentrated using a 53 µm screen filter (Florida Aqua Farms, FL, USA) down to 100 mL of culture and rinsed twice with 200 mL of ESAW medium and resuspending in 100 mL ESAW medium. Rotifers were enumerated by direct counting using a Rafter counter.

### Monitoring microalgal growth and rotifer cultures

Algal culture density was determined daily by chlorophyll fluorescence (430 nm excitation, 685 nm emission) using a Tecan i-control infinite 200Pro, version 1.11.1.0. Algal cell counts were obtained via direct enumeration with a Z2 Coulter Particle Count & Size Analyzer (Beckman Coulter, Pasadena CA).or derived by calculation via a standard curve correlating chlorophyll fluorescence with algal density. Duplicate cell counts and fluorescence measurements for each sample were averaged for each timepoint and then normalized to the final concentration measurements for the *M*. *salina* control in the absence of rotifers. Health and viability of rotifers within algal cultures was monitored daily via light microscopy. Significant differences between means of healthy or infected algal cultures were compared using two-way ANOVAs with Tukey’s HSD test.

### SPME headspace sampling and GC-MS data acquisition

VOCs were sampled in duplicate from the headspaces of each culture and medium control vessel using portable field sampler SPME fibers, with 65 µm polydimethylsiloxane/divinylbenzene (PDMS/DVB) coatings (Supelco, Bellefonte, PA). As two vessels were prepared for each condition, duplicate SPME samplings generated four replicate measurements. For this work, we required a “portable field sampler” fiber design to facilitate sample collection, transport, and storage over the timecourse of the experiments. The bi-phasic coating (one of three commercially available field-portable options) was chosen for sampling a wide range of compounds, including polar analytes, semi-volatiles, and larger weight volatiles. SPME samples were obtained within 1–2 hours of the fluorescence measurements that were used to determine algae concentrations. SPME exposure times were shorter for Experiment 1 (30 min) compared to Experiments 2 and 3 (60 min). SPME fibers were stored in refrigerators at 2–4 °C after sampling. Unexposed SPME fibers served as “travel blanks” to account for extraneous volatiles arising from storage conditions. Samples were analyzed by GC-MS within 2 weeks of collection.

An untargeted GC-MS approach was used to analyze the collected VOCs with an Agilent 5975 T GC-MSD (Agilent Technologies, Santa Clara, CA) using an Agilent HP-5ms column (30 m × 250 µm × 0.25 µm) coupled to a single quadrupole mass analyzer with helium carrier gas at a constant flow rate of 1.2 mL/min. VOCs absorbed on the SPME fiber were desorbed in the heated GC inlet (280 °C) for 15 seconds using splitless injection. The column temperature was programmed, starting at 40 °C for 3 min, ramped at 5 °C/minute from 40 to 150 °C, ramped at 15 °C/min from 150 to 280 °C and held for 2 min. The total run time was 35.67 min. Ions were generated using electron ionization (70 eV) and acquired at 4 scans/s over *m/z* 35–450. Data acquisition was performed under control of ChemStation software (Agilent Technologies, version E.02.02). A commercial reference of 18 standard compounds (S-22329; AccuStandard, New Haven, CT) was used to evaluate day-to-day performance and to calculate retention indices.

### GC-MS data processing

After GC-MS data acquisition, data processing procedures and criteria were applied to detect and identify individual biomarkers in each condition. All ChemStation data files (consisting of biological duplicates, media controls, and unexposed fibers) were translated for compatibility with Agilent’s MassHunter Software (MassHunter GC/MS Translator B.07.05). Chromatographic deconvolution and visualization were performed using MassHunter Qualitative (version B.07.00 SP2) using a Retention Time window size factor of 90.0, signal-to-noise ratio threshold of 2.00, absolute ion height filter of 1000 counts, and ≥5 ions required for compound detection (threshold of detection 5 × 10^3^ counts per peak). An arbitrary small value of 1 was assigned to the signal value for compounds that were not detected.

Detected peaks were transferred into Mass Profiler Professional (MPP) 12.6.1 software and aligned across all samples in the data set using a retention time tolerance of 0.15 minutes, mass spectral match factor of 0.6 (of maximum 1.0), and a delta *m/z* tolerance of 0.2 Da. Putative identification of the aligned compounds was performed by searching spectra against the National Institute of Standards and Technology (NIST) mass spectral database, NIST14. Compounds with mass spectral matches ≥70% were subsequently annotated as the best match. Compounds that did not exceed the mass spectral match threshold were annotated using the base peak *m/z* and retention index (e.g. “Unknown *m/z* ##_RI ####”).

Two criteria were used to identify volatile biomarkers unique to the Algae or Algae + Rotifer conditions: (1) detection of the biomarker in at least three of the four replicates at each sampled timepoint and (2) a) the biomarker was present in the Algae or Algae + Rotifer condition and absent in the media blank or travel blank conditions; OR b) the biomarker was present in the Algae or Algae + Rotifer conditions at an abundance greater than 10x the abundance in the media blank or travel blank.

The peak areas of potential biomarkers passing the filter criteria were compared across the three performed experiments, with regards to both individual biomarkers and groups of biomarkers belonging to the same compound class. The presence or absence of these biomarkers in each experiment was determined, and the calculated peak areas were compared to algal density measurements.

### Ethical approval and informed consent

No conflicts, informed consent, human or animal rights applicable.

## Supplementary information

Dataset !

## Data Availability

The datasets generated during and/or analyzed during the current study are available from the corresponding author on reasonable request.
