# Supplementary material for: Chemical Profiling of Volatile Organic Compounds in the Headspace of Algal Cultures as Early Biomarkers of Algal Pond Crashes
Source: Sci Rep. 2019 Sep 25;9:13866. doi: 10.1038/s41598-019-50125-z (PMC6761164; doi:10.1038/s41598-019-50125-z)

**Supplemental Data File**

**Chemical Profiling of Volatile Organic Compounds in the Headspace of Algal Cultures as Early Biomarkers of Algal Pond Crashes**

Kristen L. Reese^1,2^, Carolyn L. Fisher^3^, Pamela D. Lane^3^, James D. Jaryenneh^3^, Matthew W. Moorman^4^, A. Daniel Jones^2,5^, Matthias Frank^1^, and Todd W. Lane^2^*

^1^Biosciences and Biotechnology Division, Physical & Life Sciences Directorate, Lawrence Livermore National Laboratory, Livermore CA 94550, USA

^2^Department of Chemistry, Michigan State University, East Lansing, MI, 48823 USA

^3^Systems Biology Department, Sandia National Laboratories, PO Box 969, Livermore, CA 94551 USA
^4^Nano and Micro Sensors Department, Sandia National Laboratories, PO Box 5800, MS 0892, Albuquerque, NM 87185 USA

^5^Department of Biochemistry and Molecular Biology, Michigan State University, East Lansing, MI, 48823 USA

Supplemental Table 1. List of VOCs in Individual Experiments

| **Experiment 1** |  |  |  |  |  |  |  |
| --- | --- | --- | --- | --- | --- | --- | --- |
|  |  | **Compound Number** | **Mass** | **NIST ID** | **NIST % Match** | **Experimental Retention Index** | **Theoretical Retention Index** |
|  | **Algae + Rotifer Cultures** | 1 | 208 |  |  | 1074 |  |
|  |  | 2 | 152 |  |  | 1214 |  |
|  |  | 3 | 83 | 2-Butanone, 4-(2,6,6-trimethyl-1-cyclohexen-1-yl)- | 70 | 1453 | 1433 |
|  |  | 4 | 177 | trans-.beta.-Ionone | 87 | 1506 | 1486 |
|  |  | 5 | 83 | 8-Heptadecene | 75 | 1691 | 1719 |
|  |  |  |  |  |  |  |  |
|  |  |  |  |  |  |  |  |
|  | **Algae + Rotifer AND Algae Cultures** | 1 | 57 |  |  | 1134 |  |
|  |  | 2 | 148 |  |  | 1415 |  |
|  |  |  |  |  |  |  |  |
| **Experiment 2** |  |  |  |  |  |  |  |
|  |  | **Compound Number** | **Mass** | **NIST ID** | **NIST % Match** | **Experimental Retention Index** | **Theoretical Retention Index** |
|  | **Algae + Rotifer Cultures** | 1 | 59 |  |  | 987 |  |
|  |  | 2 | 82 |  |  | 1021 |  |
|  |  | 3 | 71 |  |  | 1101 |  |
|  |  | 4 | 107 |  |  | 1181 |  |
|  |  | 5 | 121 | Phenol, 2,3,5-trimethyl- | 71 | 1190 | 1235 |
|  |  | 6 | 137 | 1-Cyclohexene-1-carboxaldehyde, 2,6,6-trimethyl- | 89 | 1209 | 1220 |
|  |  | 7 | 121 | 2-Butanone, 4-(2,6,6-trimethyl-1-cyclohexen-1-yl)- | 85 | 1443 | 1433 |
|  |  | 8 | 177 | trans-.beta.-Ionone | 93 | 1495 | 1486 |
|  |  | 9 | 57 |  |  | 1692 |  |
|  |  | 10 | 143 |  |  | 1881 |  |
|  |  | 11 | 135 |  |  | 2036 |  |
|  |  | 12 | 71 |  |  | 2115 |  |
|  | **Algae + Rotifer AND Algae Cultures** | 1 | 55 | Hexanoic acid, 2-ethyl-, methyl ester | 68 | 1031 | 1043 |
|  |  | 2 | 96 | 3-Nonenoic acid, methyl ester | 73 | 1134 | 1191 |
|  |  | 3 | 341 |  |  | 1138 |  |
|  |  | 4 | 71 |  |  | 1264 |  |
|  |  | 5 | 138 |  |  | 1338 |  |
|  |  | 6 | 73 |  |  | 1774 |  |
|  |  | 7 | 154 |  |  | 1854 |  |
|  |  | 8 | 73 |  |  | 1983 |  |
|  |  | 9 | 73 |  |  | 2079 |  |
|  |  | 10 | 192 |  |  | 2183 |  |
|  |  | 11 | 73 |  |  | 2210 |  |
|  | **Algae Cultures** | 1 | 71 |  |  | 757 |  |
|  |  | 2 | 56 |  |  | 1003 |  |
|  |  | 3 | 94 |  |  | 1207 |  |
|  |  | 4 | 91 |  |  | 1530 |  |
|  |  | 5 | 109 |  |  | 1619 |  |
|  |  | 6 | 119 | 2,4-Diphenyl-4-methyl-1-pentene | 83 | 1803 | 1846 |
|  |  | 7 | 70 |  |  | 1955 |  |
|  |  | 8 | 70 |  |  | 2252 |  |
|  |  |  |  |  |  |  |  |
|  |  |  |  |  |  |  |  |
| **Experiment 3** |  |  |  |  |  |  |  |
|  |  | **Compound Number** | **Mass** | **NIST ID** | **NIST % Match** | **Experimental Retention Index** | **Theoretical Retention Index** |
|  | **Algae + Rotifer Cultures** | 1 | 118 |  |  | 971 |  |
|  |  | 2 | 82 | Cyclohexanone, 2,2,6-trimethyl- | 79 | 1021 | 1036 |
|  |  | 3 | 55 |  |  | 1054 |  |
|  |  | 4 | 107 |  |  | 1181 |  |
|  |  | 5 | 121 |  |  | 1191 |  |
|  |  | 6 | 137 | 1-Cyclohexene-1-carboxaldehyde, 2,6,6-trimethyl- | 81 | 1209 | 1220 |
|  |  | 7 | 122 |  |  | 1379 |  |
|  |  | 8 | 121 | 2-Butanone, 4-(2,6,6-trimethyl-1-cyclohexen-1-yl)- | 76 | 1419 | 1433 |
|  |  | 9 | 327 |  |  | 1429 |  |
|  |  | 10 | 401 |  |  | 1458 |  |
|  |  | 11 | 177 | trans-.beta.-Ionone | 94 | 1495 | 1486 |
|  |  | 12 | 179 |  |  | 1522 |  |
|  |  | 13 | 158 |  |  | 1675 |  |
|  |  | 14 | 57 |  |  | 1691 |  |
|  |  | 15 | 172 |  |  | 1744 |  |
|  |  | 16 | 73 |  |  | 1744 |  |
|  |  | 17 | 150 |  |  | 1776 |  |
|  |  | 18 | 73 |  |  | 1907 |  |
|  |  | 19 | 251 |  |  | 2406 |  |
|  |  | 20 | 73 |  |  | 2422 |  |
|  |  | 21 | 149 |  |  | 2496 |  |
|  |  |  |  |  |  |  |  |
|  | **Algae + Rotifer AND Algae Cultures** | 1 | 57 |  |  | 978 |  |
|  |  | 2 | 71 |  |  | 1039 |  |
|  |  | 3 | 96 | 3-Nonenoic acid, methyl ester | 74 | 1134 | 1191 |
|  |  | 4 | 341 |  |  | 1139 |  |
|  |  | 5 | 71 |  |  | 1293 |  |
|  |  | 6 | 138 |  |  | 1338 |  |
|  |  | 7 | 71 |  |  | 1370 |  |
|  |  | 8 | 57 |  |  | 1507 |  |
|  |  | 9 | 73 |  |  | 1983 |  |
|  |  | 10 | 192 |  |  | 2112 |  |
|  |  | 11 | 192 |  |  | 2197 |  |
|  |  | 12 | 73 |  |  | 2345 |  |

Supplemental Table 2. Significant difference determination between mean levels of algal cell densities amongst replicates of Algae (*M. salina*), Algae + Rotifer (*M. salina* and *B. plicatilis*) and Media Blank, MB (ESAW) calculated by ANOVA with Tukey’s HSD test

|  | Experiment 1 | | | | |  |  | Experiment 2 | | | | |  |  | Experiment 3 | | | | |
| --- | --- | --- | --- | --- | --- | --- | --- | --- | --- | --- | --- | --- | --- | --- | --- | --- | --- | --- | --- |
|  | 24 Hours | 48 Hours | 72 Hours | 96 Hours | 120 Hours | |  | 24 Hours | 48 Hours | 72 Hours | 96 Hours | 120 Hours | |  | 24 Hours | 48 Hours | 72 Hours | 96 Hours | 120 Hours |
| MB 1 vs MB 2 | - | >0.9999 | 0.9942 | >0.9999 | >0.9999 |  |  | >0.9999 | >0.9999 | >0.9999 | >0.9999 | >0.9999 |  |  | >0.9999 | >0.9999 | >0.9999 | >0.9999 | >0.9999 |
| MB 1 vs Algae 1 | - | 0.0024 | <0.0001 | <0.0001 | <0.0001 |  |  | 0.0202 | <0.0001 | <0.0001 | <0.0001 | <0.0001 |  |  | 0.4867 | 0.0696 | <0.0001 | <0.0001 | <0.0001 |
| MB 1 vs Algae 2 | - | 0.0011 | <0.0001 | <0.0001 | <0.0001 |  |  | 0.0075 | <0.0001 | <0.0001 | <0.0001 | <0.0001 |  |  | 0.3291 | 0.0136 | <0.0001 | <0.0001 | <0.0001 |
| MB 1 vs Algae + Rotifer 1 | - | 0.0004 | 0.2087 | 0.2671 | 0.2335 |  |  | 0.0088 | <0.0001 | <0.0001 | 0.0079 | 0.0051 |  |  | 0.2848 | 0.0115 | 0.0001 | <0.0001 | <0.0001 |
| MB 1 vs Algae + Rotifer 2 | - | 0.002 | 0.6522 | 0.9602 | 0.9978 |  |  | 0.0081 | 0.0001 | <0.0001 | <0.0001 | <0.0001 |  |  | 0.2356 | 0.0086 | 0.0002 | <0.0001 | <0.0001 |
| MB 2 vs Algae 1 | - | 0.0024 | <0.0001 | <0.0001 | <0.0001 |  |  | 0.0202 | <0.0001 | <0.0001 | <0.0001 | <0.0001 |  |  | 0.4861 | 0.069 | <0.0001 | <0.0001 | <0.0001 |
| MB 2 vs Algae 2 | - | 0.0011 | <0.0001 | <0.0001 | <0.0001 |  |  | 0.0075 | <0.0001 | <0.0001 | <0.0001 | <0.0001 |  |  | 0.3286 | 0.0135 | <0.0001 | <0.0001 | <0.0001 |
| MB 2 vs Algae + Rotifer 1 | - | 0.0004 | 0.0719 | 0.2662 | 0.2335 |  |  | 0.0088 | <0.0001 | <0.0001 | 0.008 | 0.0051 |  |  | 0.2844 | 0.0114 | 0.0001 | <0.0001 | <0.0001 |
| MB 2 vs Algae + Rotifer 2 | - | 0.002 | 0.3351 | 0.9598 | 0.9978 |  |  | 0.0081 | 0.0001 | <0.0001 | <0.0001 | <0.0001 |  |  | 0.2352 | 0.0085 | 0.0002 | <0.0001 | <0.0001 |
| Algae 1 vs Algae 2 | - | 0.9996 | 0.9827 | >0.9999 | 0.9898 |  |  | 0.9987 | >0.9999 | 0.2066 | 0.9996 | 0.6884 |  |  | 0.9997 | 0.9814 | 0.8709 | 0.6052 | 0.9915 |
| Algae 1 vs Algae + Rotifer 1 | - | 0.9818 | <0.0001 | <0.0001 | <0.0001 |  |  | 0.9994 | 0.9999 | 0.9711 | 0.0148 | <0.0001 |  |  | 0.999 | 0.9722 | 0.9966 | 0.7581 | 0.1778 |
| Algae 1 vs Algae + Rotifer 2 | - | >0.9999 | <0.0001 | <0.0001 | <0.0001 |  |  | 0.9991 | 0.9868 | 0.7331 | 0.8134 | <0.0001 |  |  | 0.9964 | 0.9503 | 0.9866 | 0.0302 | 0.064 |
| Algae 2 vs Algae + Rotifer 1 | - | 0.9987 | <0.0001 | <0.0001 | <0.0001 |  |  | >0.9999 | 0.9992 | 0.0422 | 0.0069 | <0.0001 |  |  | >0.9999 | >0.9999 | 0.6082 | 0.0622 | 0.0533 |
| Algae 2 vs Algae + Rotifer 2 | - | 0.9999 | <0.0001 | <0.0001 | <0.0001 |  |  | >0.9999 | 0.9723 | 0.9268 | 0.9322 | <0.0001 |  |  | >0.9999 | >0.9999 | 0.5053 | 0.0005 | 0.0163 |
| Algae + Rotifer 1 vs Algae + Rotifer 2 | - | 0.9887 | 0.9615 | 0.7415 | 0.455 |  |  | >0.9999 | 0.9984 | 0.2879 | 0.0006 | 0.0016 |  |  | >0.9999 | >0.9999 | >0.9999 | 0.4138 | 0.9958 |

Timepoints are reported relative to the addition of algae to the growth media. Rotifers were added to each condition after the 48 hour timepoint.

“-“ indicates no measurements were taken.

Supplemental Figure 1:

1a) Compound 1: Cyclohexanone, 2, 2, 6-trimethyl-


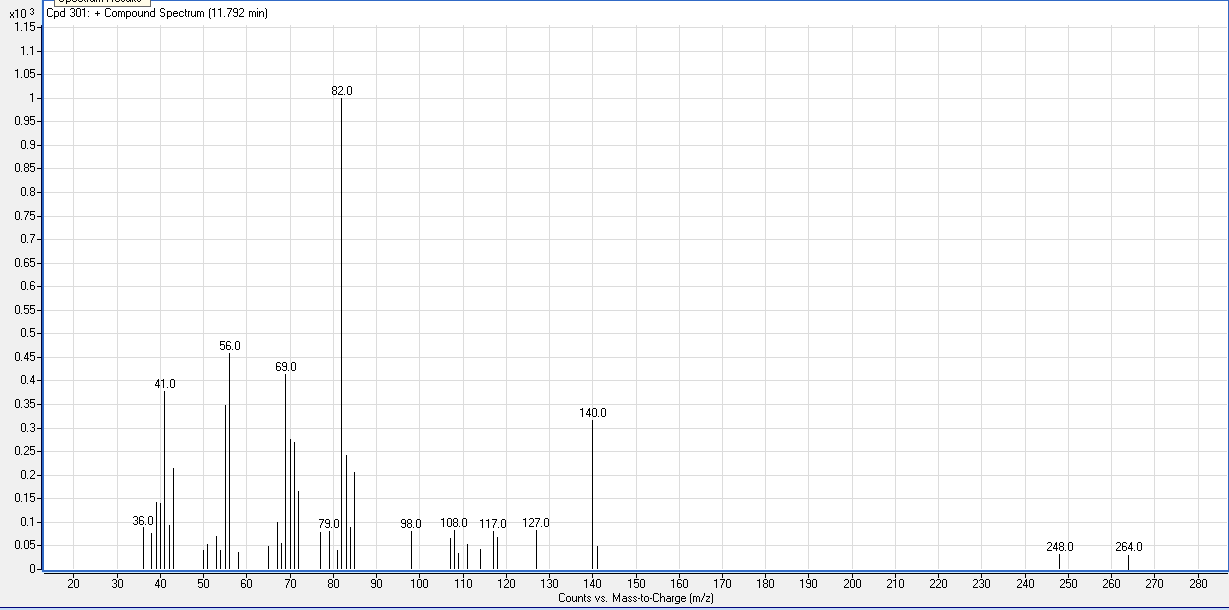


1b) Compound 2: m/z 107, RI 1181


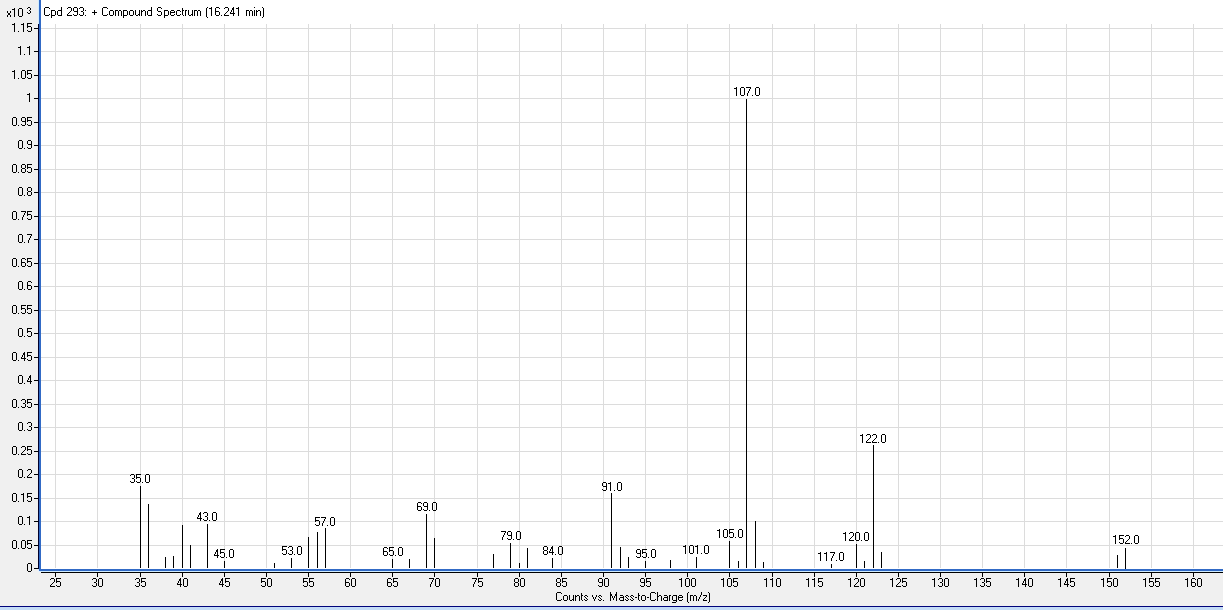


1c) Compound 3: m/z 121, RI 1191


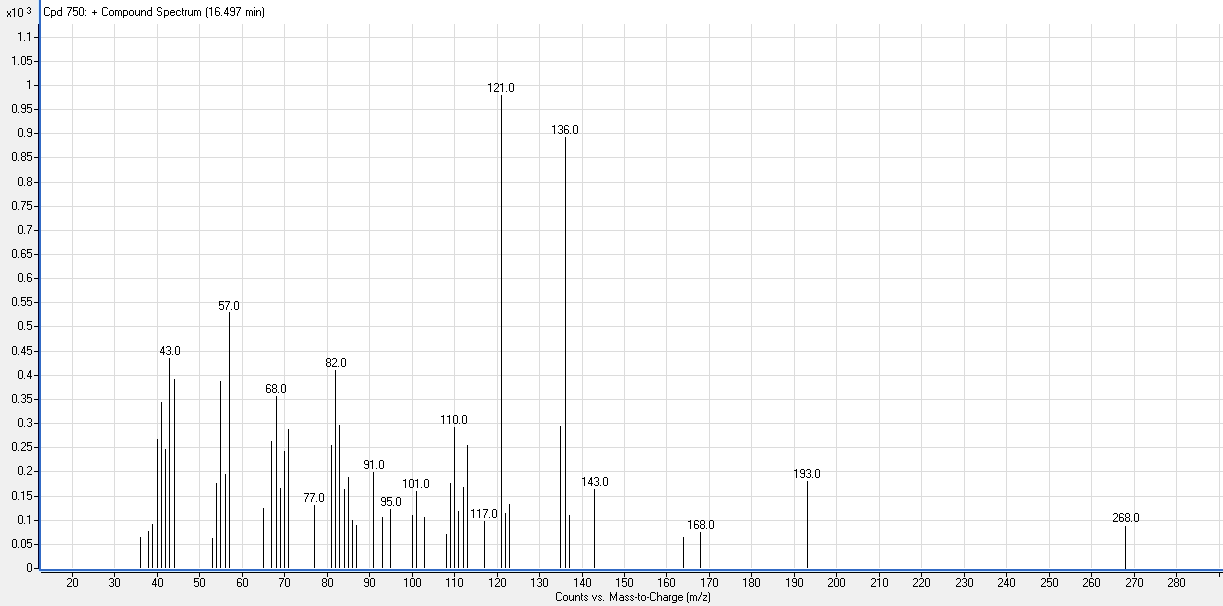


1d) Compound 4: β-cyclocitral


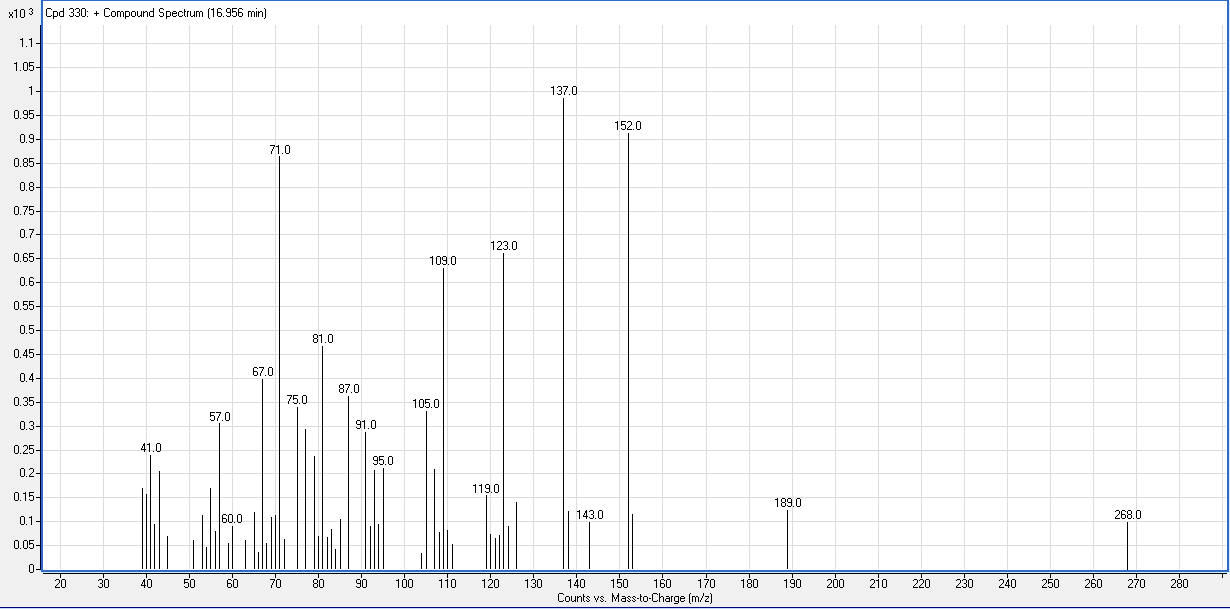


1e) Compound 5: 2-Butanone, 4-(2,6,6-trimethyl-1-cyclohexen-1-yl)-


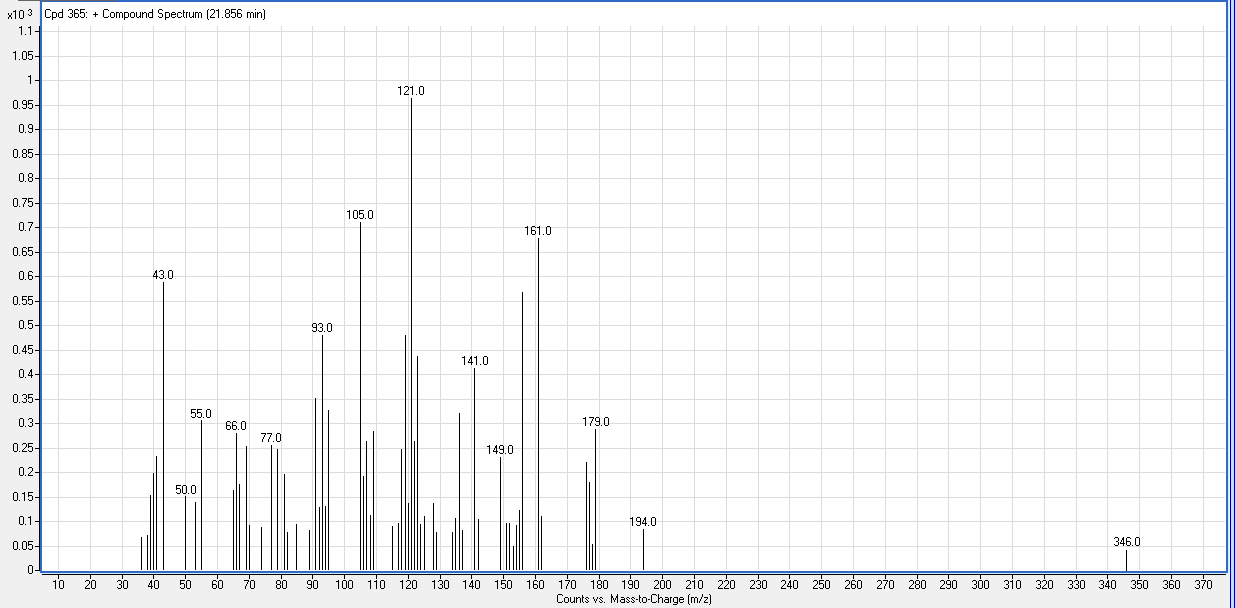


1f) Compound 6: trans-β-ionone


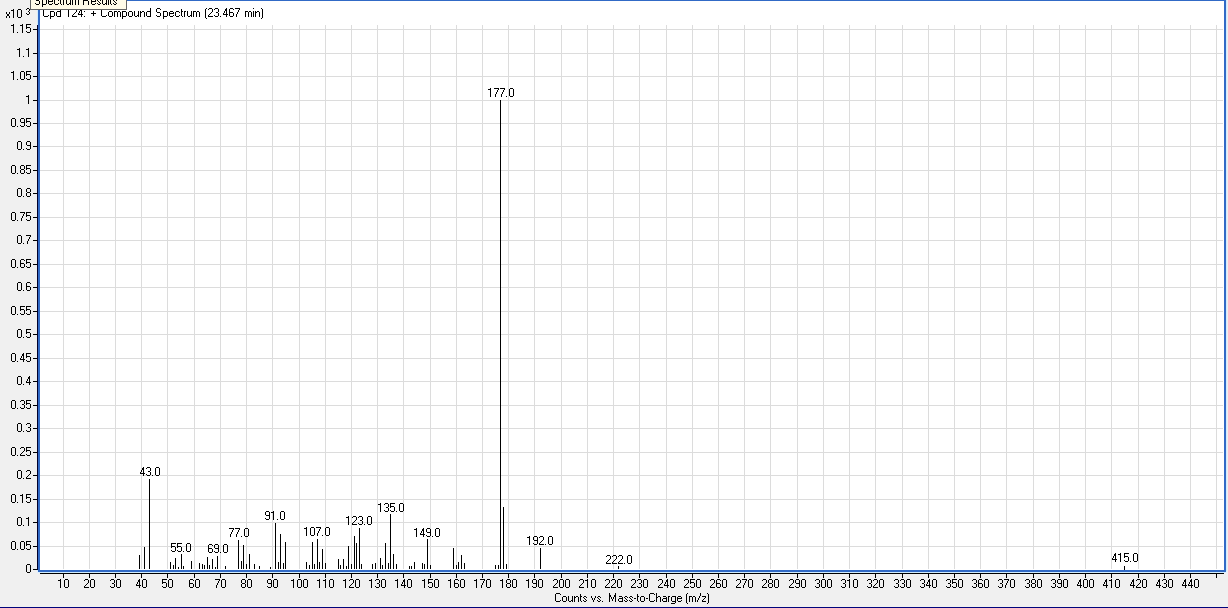


1g) Compound 7: m/z 57, RI 1691


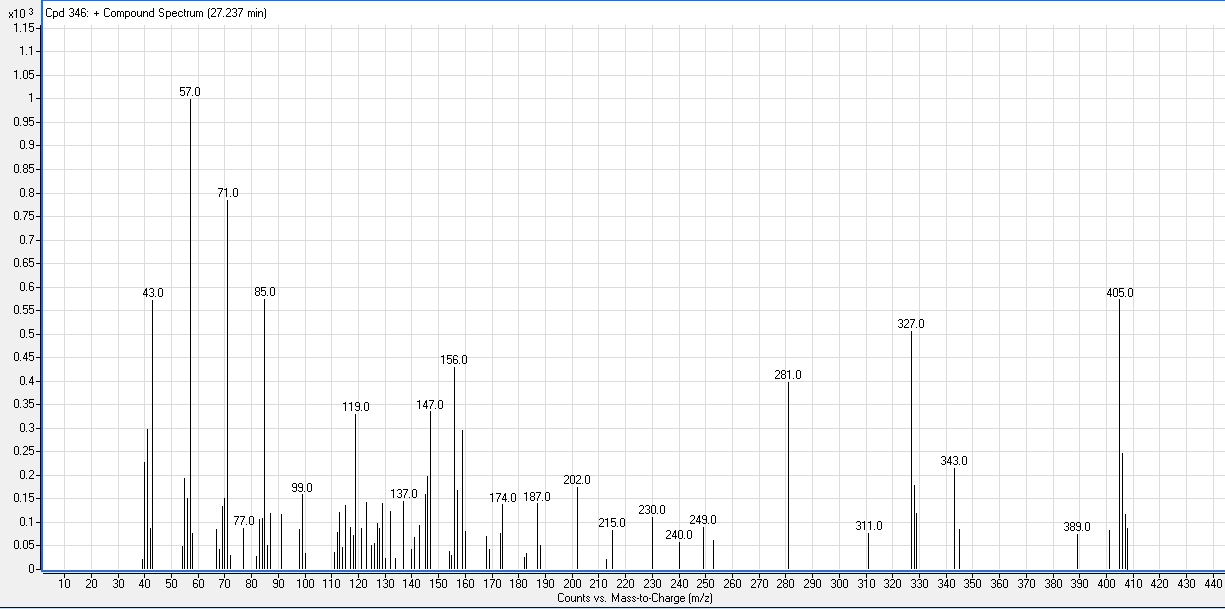


1h) Compound 8: m/z 71, RI 1039


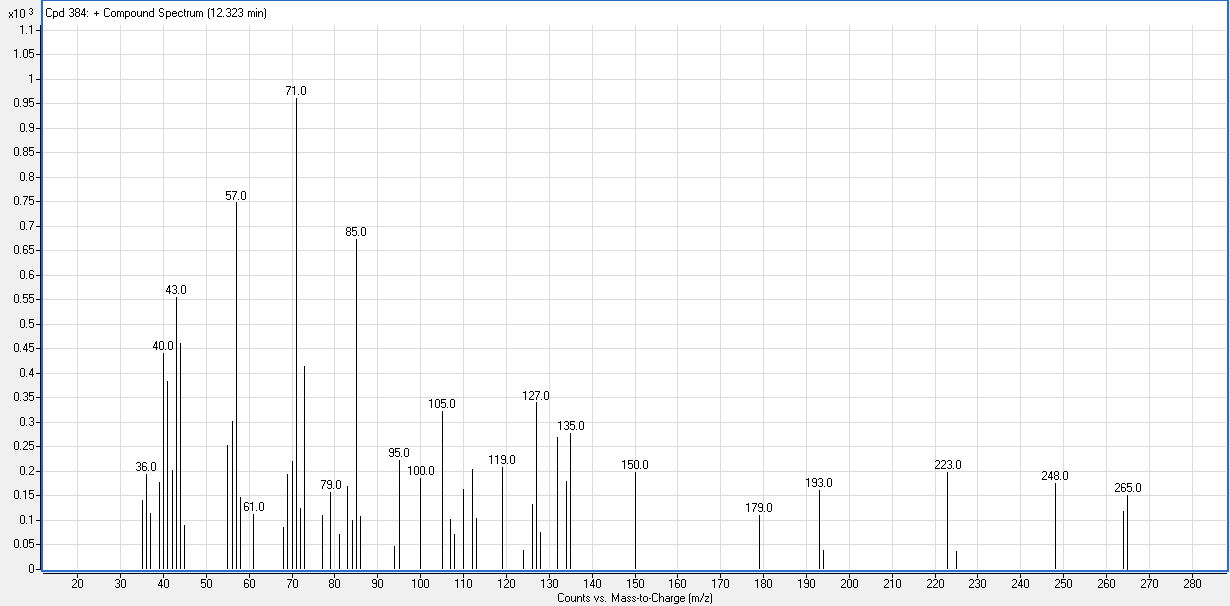


1i) Compound 9: 3-nonenoic acid, methyl ester


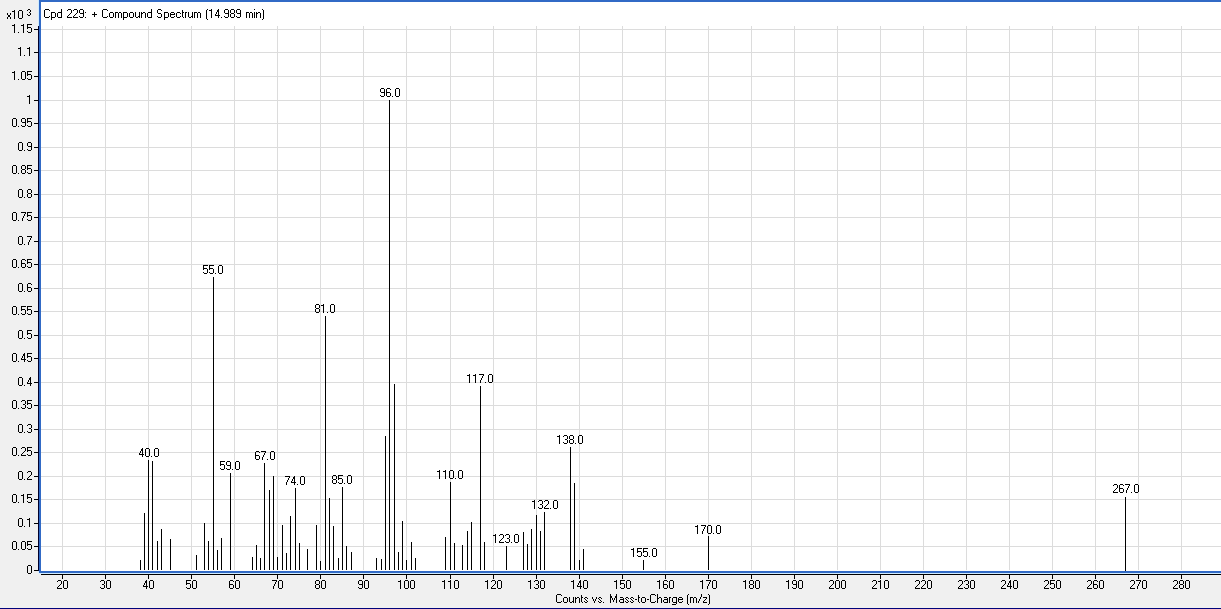


1j) Compound 10: m/z 341, RI 1139


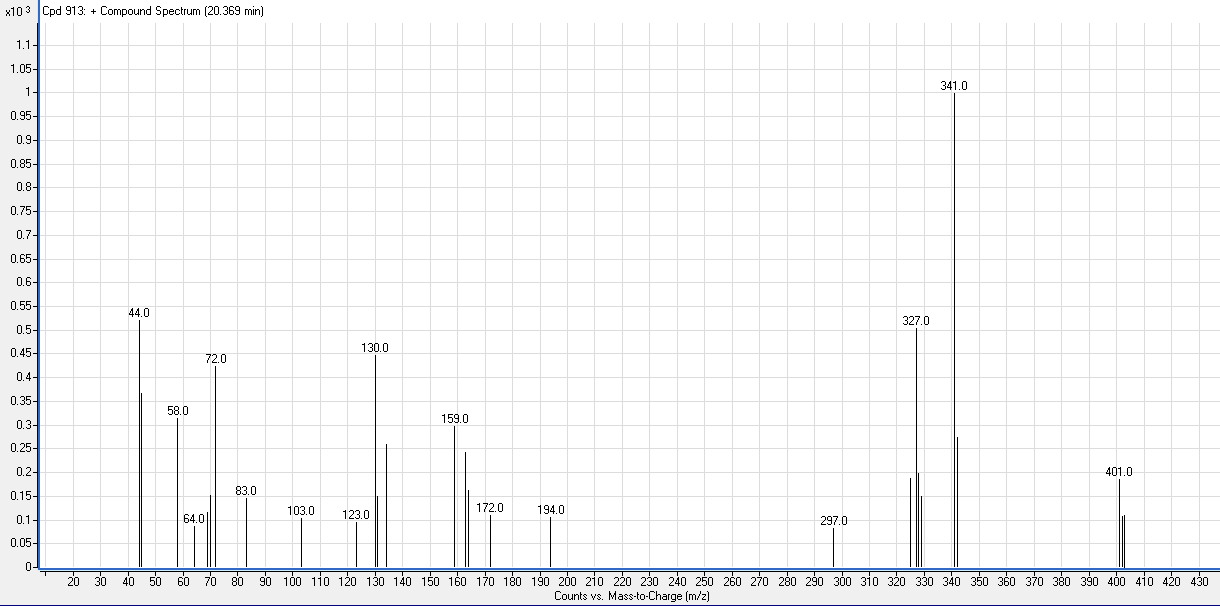


1k) Compound 11: m/z 71, RI 1293


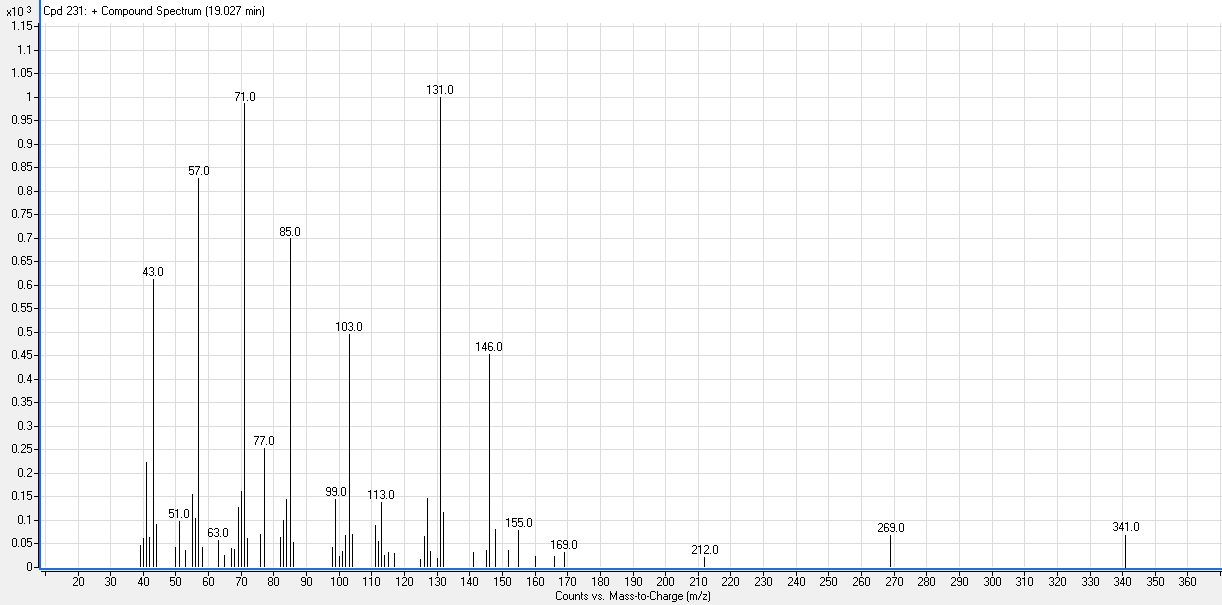


1l) Compound 12: m/z 138, RI 1338


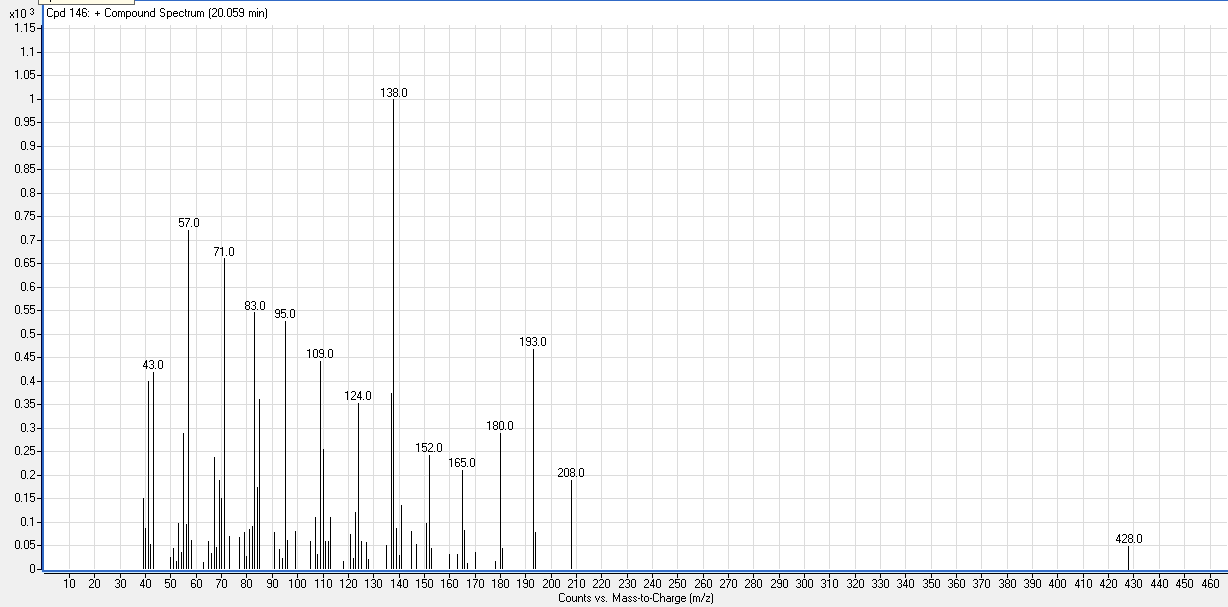


1m) Compound 13: m/z 73, RI 1983 : Hexadecanoic acid


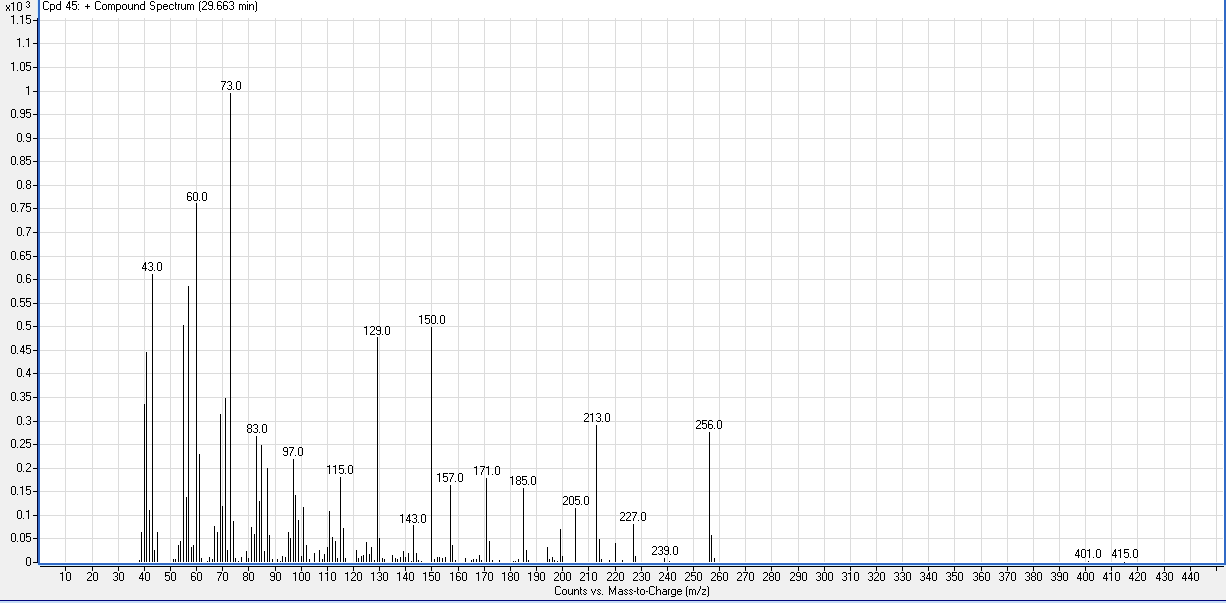


1n) Compound 14: m/z 192, RI 2197


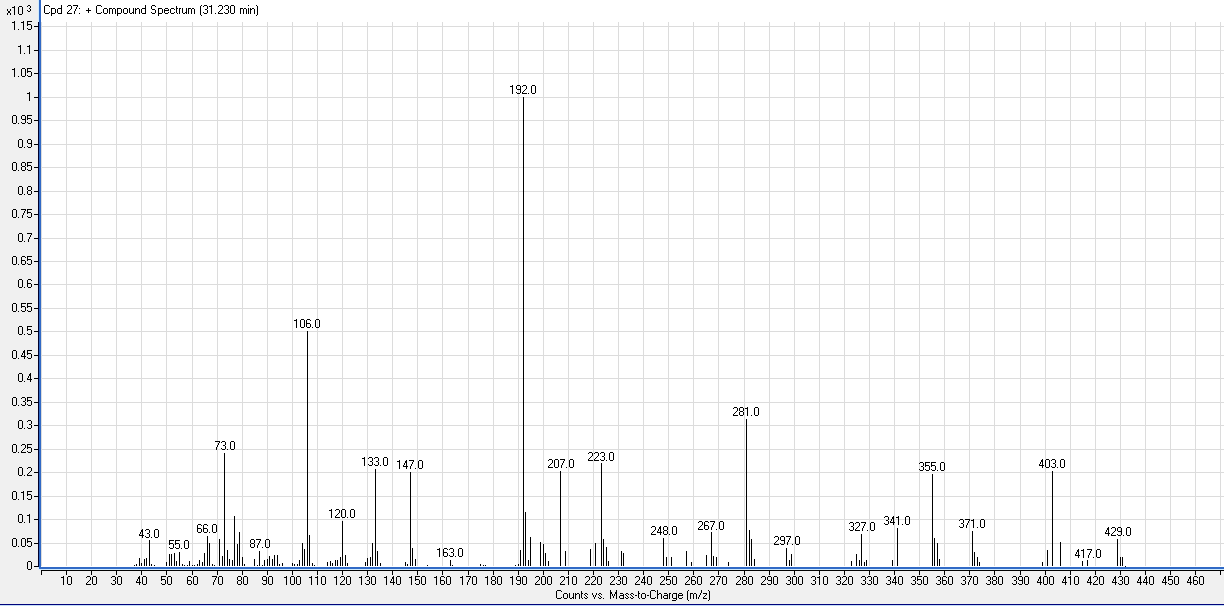


Supplemental Figure 2: Peak areas of extracted compound chromatograms for Compounds 1, 2, 3, 5, and 7 across Experiments 1, 2, and 3; Compounds 4 and 6 are displayed in Figure 5


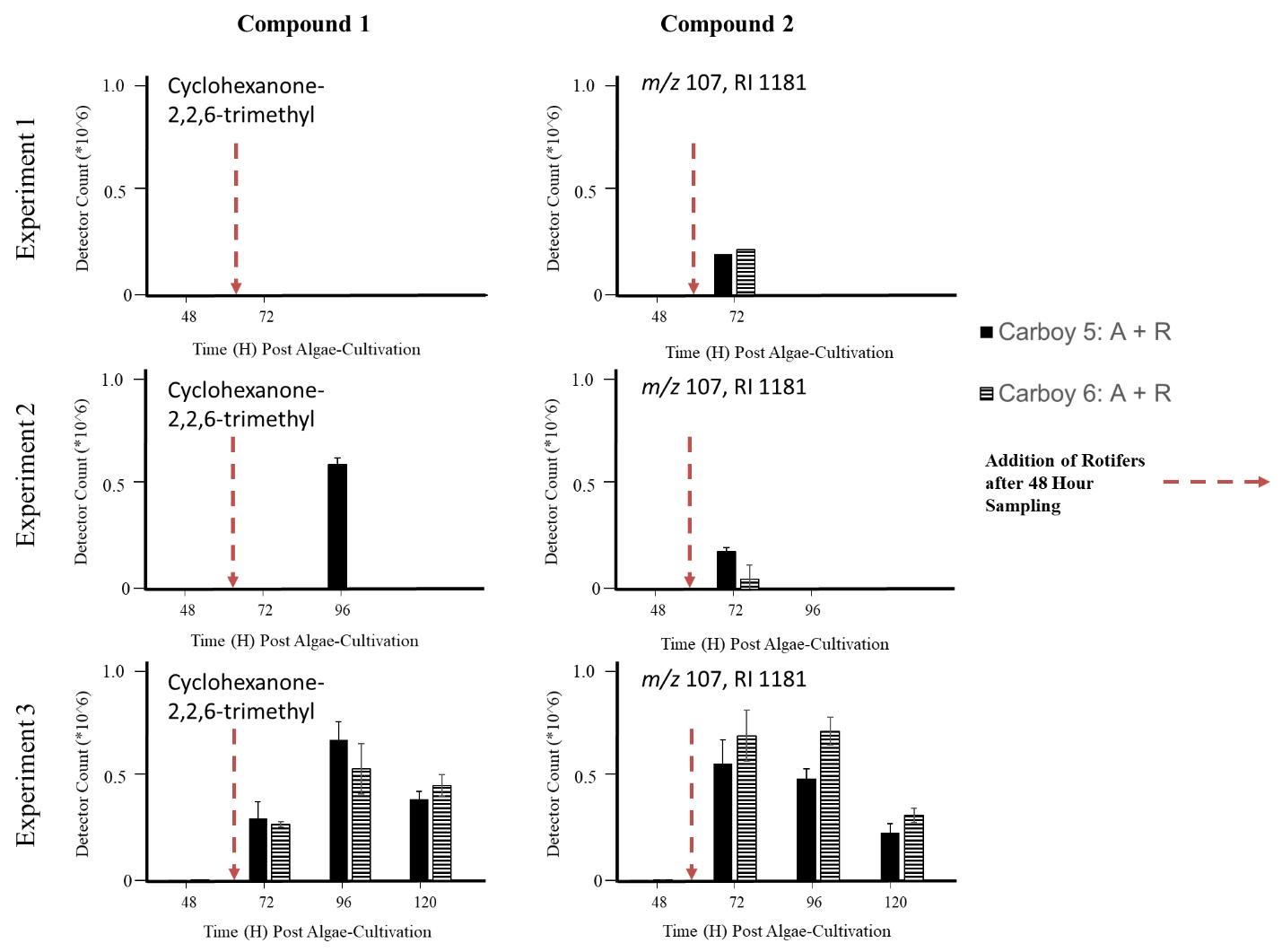


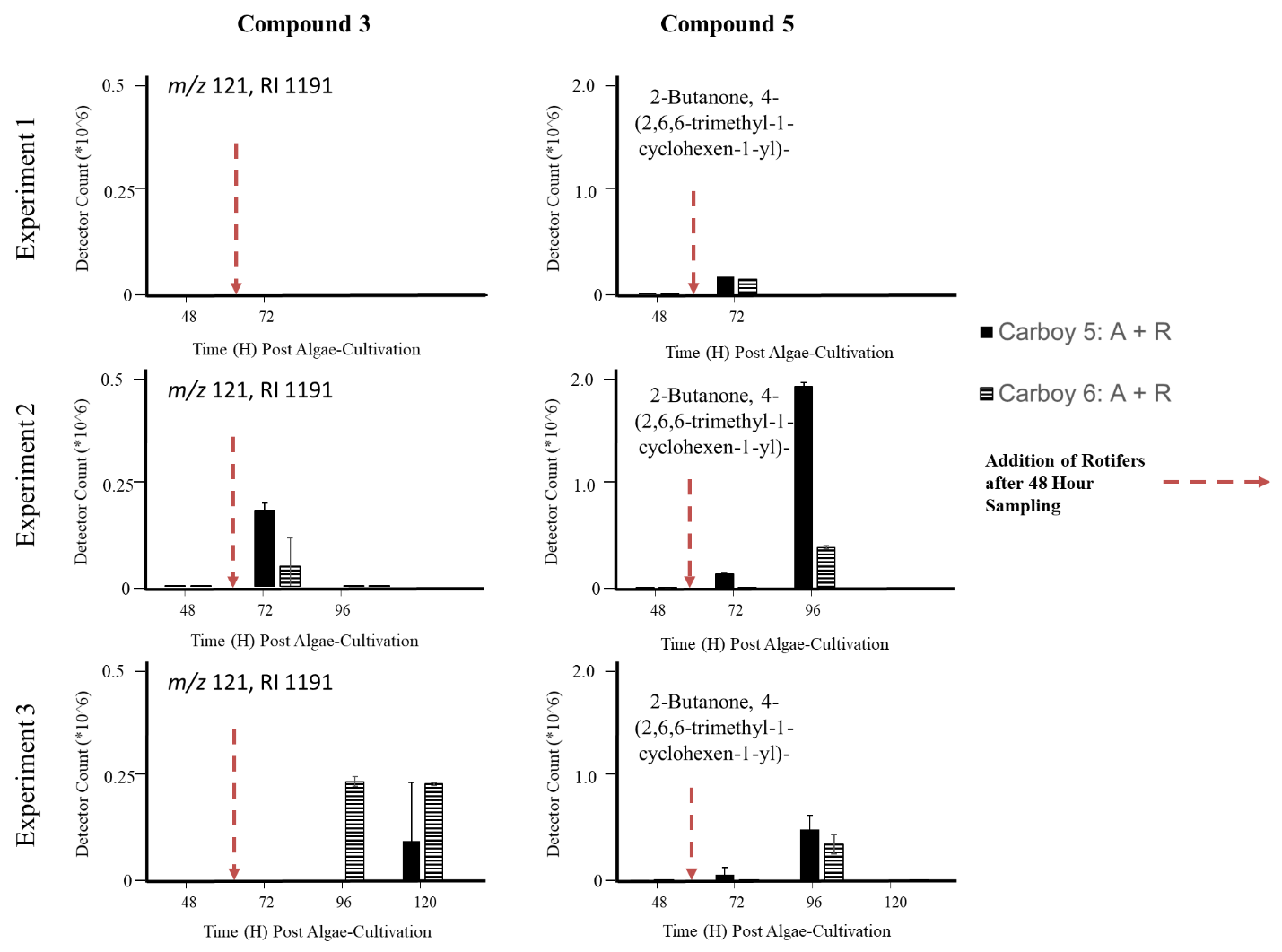


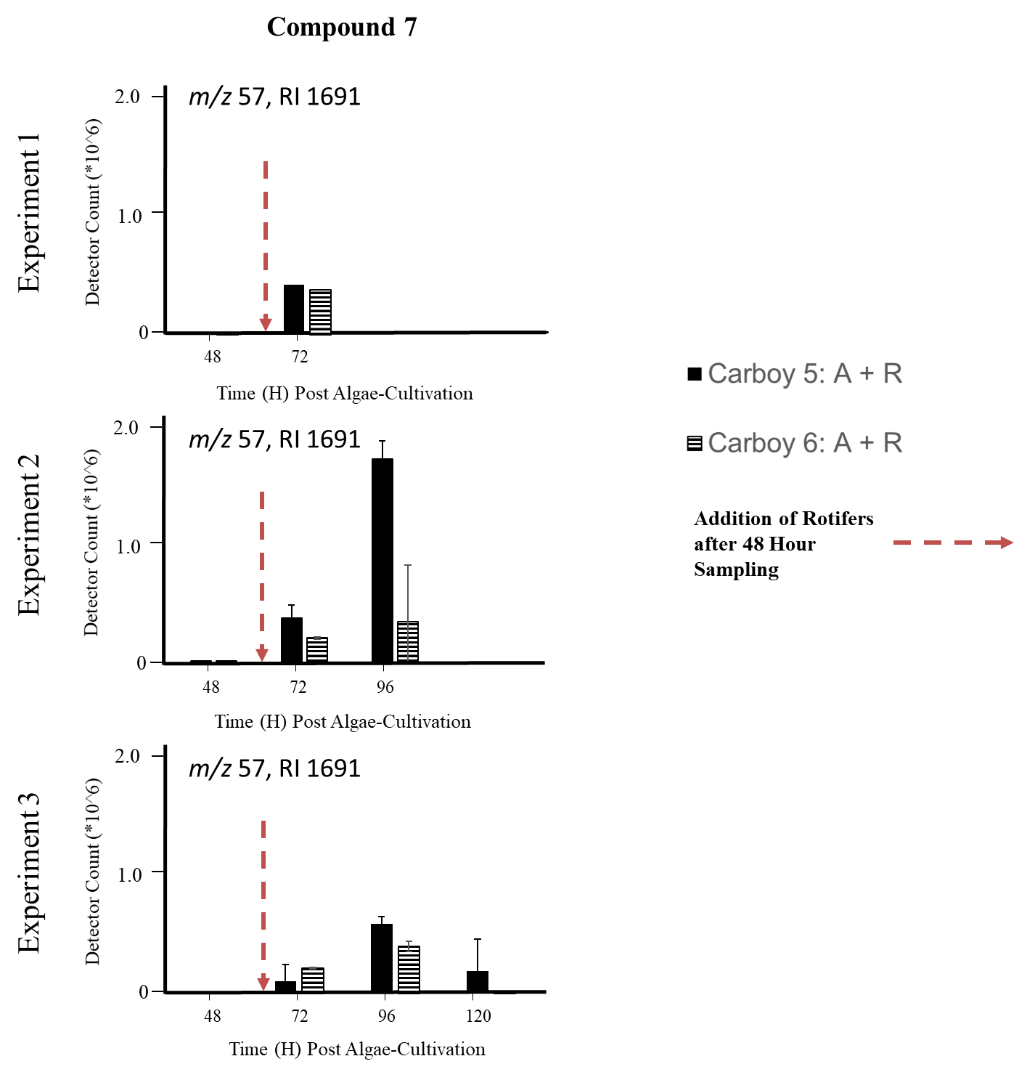

Supplement: Supplementary file 1 — Dataset ! [file 41598_2019_50125_MOESM1_ESM.docx]
